# Case Report: Sclerosing variant of extramedullary hematopoietic tumor primary arising in kidney, unexpected clinical and pathological presentation

**DOI:** 10.3389/fonc.2025.1573784

**Published:** 2025-05-12

**Authors:** Haneen Al-Maghrabi, Ghadeer Mokhtar, Feras Asali, Abdelrazak Meliti

**Affiliations:** Department of Pathology and Laboratory Medicine, King Faisal Specialist Hospital and Research Center, Jeddah, Saudi Arabia

**Keywords:** extramedullary hematopoiesis, sclerosing variant of EMH, EMH, kidney, lymphoma

## Abstract

Extramedullary hematopoiesis (EMH) is a physiological process that occurs outside the bone marrow, often in response to various hematological conditions. The sclerosing variant of EMH in the kidney is a rare manifestation characterized by the proliferation of hematopoietic cells within the renal interstitium, accompanied by significant fibrous tissue formation. This report presents a case of a patient with chronic myelofibrosis who exhibited the sclerosing variant of EMH. Imaging studies revealed renal masses, which were initially misinterpreted as malignant lesion. Histopathological examination of the renal tissue demonstrated a marked increase in hematopoietic cell types, including myeloid and erythroid lineages, interspersed with abundant collagenous stroma indicative of sclerosis. The clinical implications of this variant are noteworthy, as it may mimic renal tumors and lead to unnecessary surgical interventions. Understanding the histopathological characteristics and clinical context of sclerosing EMH in the kidney is crucial for accurate diagnosis and management. This case underscores the importance of considering EMH in the differential diagnosis of renal masses, particularly in patients with underlying hematological disorders or chronic renal conditions. Further research is needed to elucidate the pathophysiological mechanisms driving this variant and its potential implications for renal function and overall patient outcomes.

## Introduction

Hematopoiesis is a continuous process that involves the production of blood and immune cells through the activity of hematopoietic stem cells (HSCs). These versatile cells have the unique ability to both self-renew and differentiate, ultimately giving rise to a diverse range of mature cell types, including red blood cells, T-cells, B-cells, monocytes, and neutrophils (1). A vital aspect of hematopoiesis is the niche that governs the self-renewal and differentiation of HSCs, which is essential to produce blood cells. The niche comprises both hematopoietic and non-hematopoietic lineages that fulfill distinct yet occasionally overlapping functions. Although hematopoiesis mainly takes place in the bone marrow of adult, it can also occur in extramedullary locations during periods of stress in the organism to boost or maintain hematopoietic production—a process referred to as extramedullary hematopoiesis (EMH) (2). Sclerosing variant of extramedullary hematopoietic tumor (SEMHT) is an uncommon lesion that can occur in individuals with chronic myeloproliferative disorders (CMPDs), particularly in cases of idiopathic myelofibrosis accompanied by myeloid metaplasia (3). Morphologically, these lesions consist of large, atypical megakaryocytes distributed throughout a stroma that is marked by dense collagen fibrosis or myxoid alterations. The site of the tumor, the patient’s age, and the histological characteristics could indicate a diagnosis of sarcoma, carcinoma, or Hodgkin lymphoma. This report describes a case of SEMHT identified after a right nephrectomy, which was initiated due to MRI findings suggesting the presence of transitional cell carcinoma.

## Case presentation

**Figure 1:**
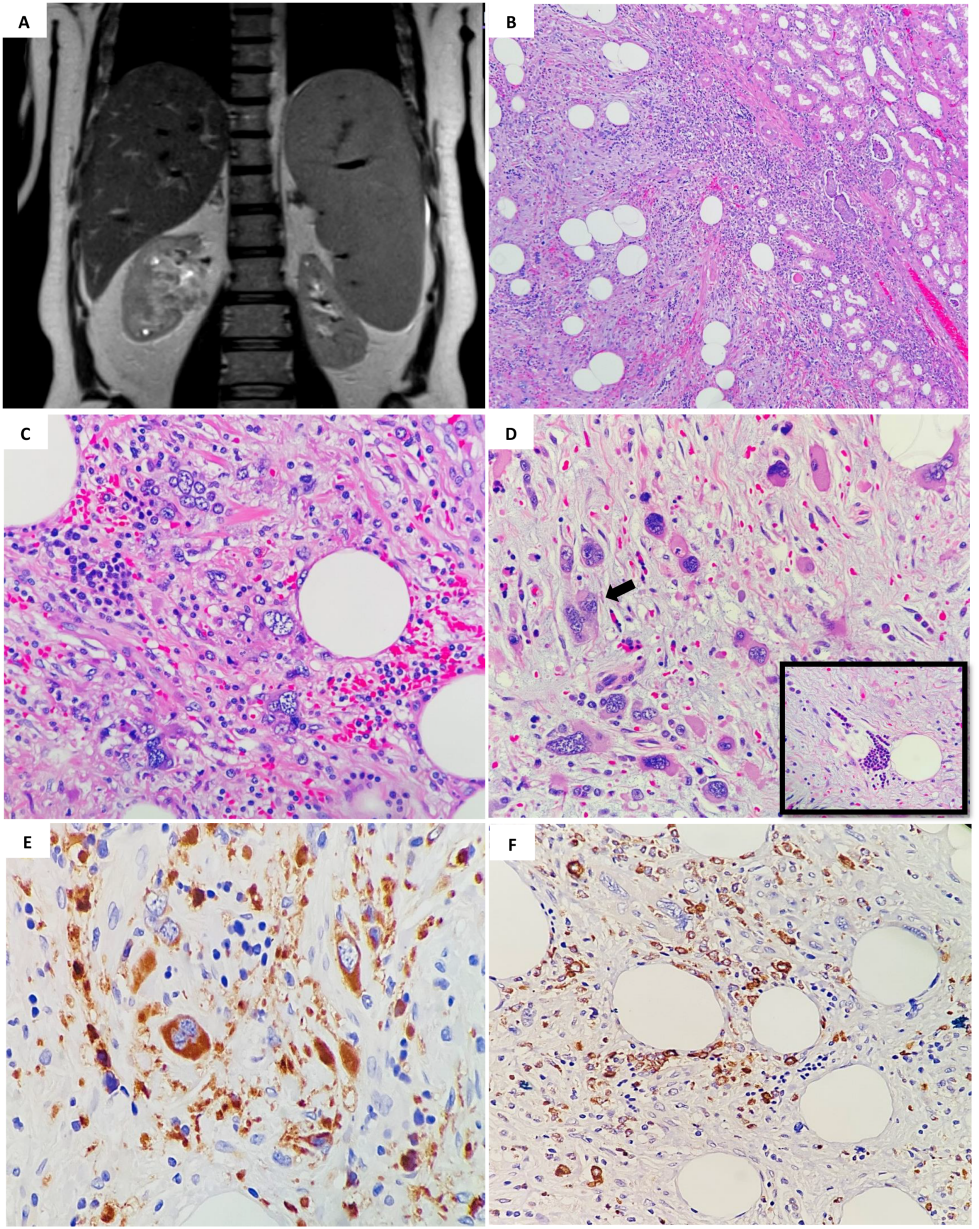
**(A)** MRI of abdomen showed 5 x 3 cm mass in the right renal hilum with heterogeneous signal intensity, extending into the lower polar and interpolar calices as well as into the right renal pelvis. **(B)** Histopathology examination by hematoxylin and eosin stain (H&E); showed a low- power magnification of the well demarcated lesion in relation to adjacent renal parenchymal tissue (H&E; 4x). **(C)** Increased numbers of hematopoietic cells are present admixed with mature fatty tissue (H&E; 40x). **(D)** Pleomorphic megakaryocytes (arrow) and groups of erythroid and myeloid precursors (insert) that represent three hematologic precursor cell-lines, are seen with a sclerotic stroma (H&E; 40x). **(E)** Immunohistochemical staining using CD61 reveals positive megakaryocytes in extramedullary hematopoiesis (stained brown color) (×40). **(F)** MPO positively highlighting the immature erythroid and myeloid precursors (×40).

A 56-year-old male patient, case of chronic renal failure with high serum creatinine level, diagnosed with primary myelofibrosis JAK2 mutation positive for more than 10 years ago. His bone marrow biopsy at the time of diagnosed showed finding consistent with primary myelofibrosis (pre-fibrotic stage). A repeat bone marrow biopsy after four months showed myeloproliferative neoplasm in favor of chronic myelofibrosis, fibrotic stage with no evidence of progression to acute myeloid leukemia. Three years prior to presentation he noticed to have splenomegaly with leucoerythroblastic picture in peripheral bone marrow, and bone marrow biopsy showed post–polycythemia vera myelofibrosis with diffuse fibrosis, grade III. His dynamic international prognostic scoring system (DIPSS) scored intermediate-1. The patient was controlled on hydroxyurea and aspirin. He came to urology clinic complaining of mild right sided flank pain. The pain was on and off, moderate in severity, not associated with hematuria. Computed tomography (CT) scan performed six months before the MRI shows significant fat stranding at the renal hilum and in the proximal periureteric area. There is a soft tissue density change suggestive of fullness in the renal pelvis, but this is challenging to define due to the limitations of a non-contrast study. The left kidney is slightly malrotated, facing forward, and there are no signs of renal stones or hydronephrosis. The urinary bladder appears collapsed without any intravesical stones. The absence of IV contrast restricted the assessment of the solid organs. The liver is enlarged, measuring 19.5 cm, with a homogenous appearance and no clear focal lesions or intrahepatic biliary dilation. The spleen is also enlarged, measuring 16.5 cm in its largest craniocaudal dimension. The pancreas, both adrenal glands, and pelvic organs appear grossly normal. Standard magnetic resonance imaging (MRI) of abdomen with the renal protocol showed 5 x 3 cm mass in the right renal hilum with heterogeneous signal intensity. The mass was extending into the lower polar and interpolar calices as well as into the right renal pelvis. No local lymphadenopathy and nor urolithiasis. The left kidney appears unremarkable without any hydronephrosis or mass. The overall findings were most in keeping with transitional cell cancer involving the right collecting system (**Figure 1A**). Also, the radiological studies indicated persistent hepatic and splenomegaly, with no focal lesions. The patient was admitted for right robotic nephrectomy and the resected kidney was sent for histopathology evaluation. Gross evaluation reveals a poorly defined firm, yellow-tan lesion with area of hemorrhage extending into and obliterating the renal pelvis measuring 7 cm in maximum dimension. Histopathology sections showed hematopoietic elements including megakaryocytes, myeloid and erythroid components within background of adipose tissue and fibromyxoid stroma (**Figure 1B**). There are scattered large degenerated and hyperchromatic nuclei appreciated but no evidence of atypical mitosis or necrosis (**Figures 1C, D**). The above findings are consistent with the rarely described SEMHT arising in the kidney. Surgical resection margins were negative. Immunohistochemistry stains supports the above diagnosis, with CD61 expression by megakaryocytes (**Figure 1E**) while myeloperoxidase (MPO) (**Figure 1F**) and factor VIII expressed in other hematopoietic elements. While pancytokeratin, desmin, CD30, CD15, CD117, and S-100 all were negative. The entire lesion was submitted for pathological examination and reprocessed. Additionally, a consultation was obtained from a specialized hematopathologist, which confirmed the final diagnosis of SEMHT. The patient was discharged without any postoperative complications and is following up regularly with the hematology clinic for three months follow up.

## Discussion

CMPDs are the result of the clonal expansion of myeloid stem cells. Bone marrow fibrosis, primarily associated with chronic idiopathic myelofibrosis featuring myeloid metaplasia, can also occur in any CMPD. It arises from the proliferation of nonclonal fibroblasts, which appears to be triggered by the inappropriate release of growth factors from clonal megakaryocytes or platelets (4). Cytokines associated with the progression of myelofibrosis encompass transforming growth factor-β, epidermal growth factor, platelet-derived growth factor, basic fibroblast growth factor, and calmodulin (5). These factors facilitate the migration and proliferation of fibroblasts, encourage angiogenesis, and enhance the accumulation of various extracellular matrix proteins, including interstitial and basement membrane collagens, fibronectin, laminin, vitronectin, and tenascin (6). These growth factors derived from megakaryocytes probably play a role in the formation of SEMHT, although the specific mechanism behind localized tumor growth remains unclear. SEMHTs are often observed in CMPD that have persisted for a long time, suggesting that they may indicate a progression to advanced disease (7). Patients often experience unfavorable outcomes, likely stemming from the advanced myelofibrosis rather than the presence of the SEMHT. Additionally, due to the link between SEMHT and CMPD, these lesions are more commonly seen in older patients (8). The ages of patients at the time of diagnosis varied from 41 to 75 years, with a median age of 65.5 years. SEMHHT typically appear as a solitary mass or multiple masses, and have been identified in various locations, such as the pleura, lung, intestinal mesentery, abdominal peritoneal lining (7), and the orbital cavity surrounding the eye (8, 9). Notably, these tumors most frequently occur in the retroperitoneal area (8). An analysis of the literature indicated that 11 out of 23 cases (48%) occurred in a retroperitoneal location. Among these, 2 cases showed direct involvement of the renal capsule, 1 case involved the renal pelvis without affecting the kidney, and 2 cases demonstrated considerable involvement of the renal parenchyma (3, 7, 8). Additionally, we found a few rare case reports of SEMHHT affecting the kidney (10, 11).

The presence of a prominent fibrotic component and atypical megakaryocytes in SEMHT can lead to misdiagnosis as other types of tumors, including sclerosing liposarcoma, rhabdomyosarcoma, carcinoma, Hodgkin’s lymphoma, and myelolipoma, particularly if the clinical context is not considered. SEMHTs may exhibit several characteristics like sarcomas, particularly sclerosing liposarcomas. Both SEMHTs and sclerosing liposarcomas typically occur in older individuals and are often found in the retroperitoneal space. These tumors consist of alternating regions of mature adipose tissue and fibrous or collagenous stroma that contain numerous large, atypical, bizarre cells, which are predominantly dispersed as isolated cells. Both tumors can contain areas of small lymphocytes and plasma cells; however, certain characteristics can aid in differentiating SEMHTs from sclerosing liposarcomas. On gross examination, SEMHTs may present as single tumors or as multiple nodules, whereas sclerosing liposarcomas typically occur as solitary mass. Morphologically, SEMHTs exhibit trilineage hematopoiesis characterized by the presence of large, atypical megakaryocytes, while sclerosing liposarcomas are composed of large abnormal cells without any hematopoietic components. Immunohistochemical analysis involving antibodies specifically targeting antigens associated with the three myeloid cell lines (e.g. myeloperoxidase for granulocytes, hemoglobin for erythroid cells, and factor VIII for megakaryocytes) could aid in identifying the presence of extramedullary hematopoiesis in SEMHT. On the other hand, large, atypical cells in sclerosing liposarcomas typically express S-100 protein. In addition, in sclerosing liposarcomas, tumor cells can invade the adjacent vascular walls. In contrast, while SEMHTs may feature prominent thick-walled blood vessels, which are not infiltrated by tumor cells (12). The differential diagnosis for SEMHT also includes Hodgkin’s lymphoma, particularly the lymphocyte depletion type (LDHD). Both SEMHT and LDHD are typically seen in older patients and can lead to hepatosplenomegaly. Morphologically, LDHD, like SEMHT, is often hypocellular and exhibits a diffusely fibrotic background, frequently containing trapped lipocytes. While atypical megakaryocyte-like bizarre sarcomatous Reed-Sternberg cell variants may be prevalent, hematopoietic components, which are consistently present in SEMHT are lacking in LDHD. Immunophenotyping analyses can be employed to confirm the typical presentation of Hodgkin’s lymphoma or to identify hematopoietic elements in cases of SEMHT where morphological characteristics are inconclusive. Hodgkin’s cells are typically positive for CD15, CD30, PAX5 (dim staining). While negative for other markers such as CD45, CD20, CD3, CD5, factor VIII, hemoglobin, and myeloperoxidase (13). SEMHT exhibit several characteristics like those of benign myelolipomas. While myelolipomas are typically linked to the adrenal gland, they can also develop in other areas of the abdominal cavity. In a similar fashion, SEMHTs are frequently found in the intra-abdominal region and may originate in the adrenal gland or around the kidneys. Histologically, both neoplasms consist of mature adipose tissue and trilinear hematopoietic components, although the adipocytes are more prevalent in myelolipomas. A helpful distinguishing feature is the sclerotic background, which is commonly found in SEMHTs but usually missing in myelolipomas. Additionally, while both tumors demonstrate trilineage hematopoiesis, the hematopoietic components found in myelolipomas lack atypical cytological characteristics. In contrast, atypical megakaryocytes are a defining feature of SEMHTs (14).

It is important to mention that when distinguishing between EMH and clear renal cell carcinoma (CRCC) in patients with CMPD, several clinical and radiological indicators can assist in making the differentiation. EMH is closely linked to a documented history of CMPD, such as myelofibrosis. Therefore, a prior diagnosis of CMPD strongly supports the diagnosis of EMH. Patients with EMH typically exhibit systemic symptoms associated with CMPD, including splenomegaly, hepatomegaly, anemia, thrombocytopenia or thrombocytosis; these were all present in our case. Conversely, CRCC generally presents with different systemic symptoms such as hematuria, flank pain, and weight loss. Radiological imaging can support the diagnosis; EMH usually appears as homogeneous soft tissue masses on imaging studies (CT or MRI), while CRCC frequently shows heterogeneous enhancement due to areas of necrosis or hemorrhage. EMH can manifest as multiple masses, unlike CRCC, which typically presents as a single mass. Also, EMH may involve the renal parenchyma or surrounding perirenal space, whereas CRCC usually originates in the renal parenchyma. Evaluating images of the spleen and liver can provide significant insights; the presence of splenomegaly and hepatomegaly alongside renal masses strongly suggests EMH. Finally, bone marrow biopsy can significantly aid in confirming the presence of CMPD. To conclude, key differences to consider is the presence of EMH at other sites is a strong indicator in favor of EMH, the patient’s hematological profile plays a vital role in the differentiation. The pattern of enhancement seen in imaging studies can also help distinguish between the two conditions. It’s essential to convey that a definitive diagnosis usually necessitates a tissue biopsy (8).

The overall disease survival is variable. However, their unfavorable outcome is more likely due to the progression of the disease rather than the presence of SEMHT.

## Conclusion

In conclusion, we present a case of SEMHT identified in a nephrectomy specimen from a 56-year-old man who exhibited flank pain and whose radiological assessments indicated the presence of transitional cell carcinoma. We propose that this condition should be included in the differential diagnosis when a renal biopsy/tissue resection show a lesion with morphological characteristics indicative of sarcoma or carcinoma exhibiting sarcomatoid features. A tissue needle core biopsy could help prevent the patient from undergoing unnecessary surgery. SEMHT may initially indicate the presence of a malignant kidney or retroperitoneal tumor. Its histological characteristics might resemble those of sclerosing liposarcoma, sarcomatoid carcinoma, or Hodgkin’s lymphoma. For individuals with a history of CMPD, it is essential to exclude SEMHT.

## Data Availability

The original contributions presented in the study are included in the article/supplementary material. Further inquiries can be directed to the corresponding author.
